# Genetic Associations with Pectus Excavatum: A Systematic Review

**DOI:** 10.3390/cimb48010122

**Published:** 2026-01-22

**Authors:** Redoy Ranjan, Nafiz Imtiaz, Benjamin Waterhouse, Ian Paul, Annemarie Brunswicker, Joel Dunning

**Affiliations:** 1 Department of Cardiothoracic Surgery, James Cook University Hospital, Middlesbrough TS4 3BW, UK; benjamin.waterhouse2@nhs.net (B.W.); ianpaul@nhs.net (I.P.); annemarie.brunswicker1@nhs.net (A.B.); joeldunning@nhs.net (J.D.); 2 Department of Biological Sciences, Royal Holloway University of London, London TW20 0EX, UK; 3 Department of Psychiatry, North East London NHS Foundation Trust, London RM13 8GQ, UK; 4 South Tees Hospitals NHS Foundation Trust, Middlesbrough TS4 3BW, UK

**Keywords:** pectus excavatum, genetics, genome-wide association study, GWAS, chest wall deformity, collagen, cartilage, systematic review

## Abstract

Background: Pectus excavatum (PE) is the most common congenital chest wall deformity, affecting approximately 1 in 400 live births. Although familial clustering supports a genetic contribution, the molecular basis of PE remains poorly defined. This systematic review synthesizes existing evidence on genetic variants associated with PE to guide future genome-wide association studies (GWAS) and Mendelian randomization (MR) analyses. Methods: A comprehensive systematic search was conducted across all electronic databases, including Google Scholar, PubMed/MEDLINE, Web of Science, and arXiv, from inception to November 2025. Nine studies met the inclusion criteria. The search strategy utilized the terms “pectus excavatum”, “genetic variants”, “SNPs”, and “GWAS”, combined with Boolean operators. Eligible studies reported genetic associations, family-based analyses, or mechanistic investigations. The Newcastle–Ottawa Scale was used to assess study quality. Results: No population-level GWAS of isolated PE was identified. Fourteen genetic loci were reported across diverse study designs, including family-based exome sequencing (*REST*, *SMAD4*, *COL5A1*, *COL5A2*), case reports (*COL1A1*, *COL27A1*, *NF1*, *BICD2*, *PTPN11*), candidate gene analyses (*ACAN*), mouse models (*GPR126*, *GAL3ST4*), and linkage analysis implicating chromosome 18q. These genes converge on four key biological pathways: extracellular matrix and collagen metabolism, TGF-β/BMP signaling, cartilage development, and transcriptional regulation. Importantly, none of the included studies reported SNP-level effect sizes, allele frequencies, or odds ratios, precluding construction of valid MR instruments. Conclusions: Current genetic evidence for PE is largely derived from rare variants and family-based studies, with no population-level GWAS available. This critical gap limits causal inference, underscoring the urgent need for large-scale international GWAS to identify common variants and clarify the genetic architecture of PE.

## 1. Introduction

Pectus excavatum (PE), also known as funnel chest, is the most prevalent congenital chest wall deformity, characterized by posterior depression of the sternum and adjacent costal cartilages [1]. With an estimated prevalence of 1 in 300 to 1 in 400 live births and a male-to-female ratio of approximately 3:1, PE represents a significant clinical entity affecting both pediatric and adult populations [2,3]. The condition ranges from mild cosmetic concerns to severe cardiopulmonary compromise requiring surgical intervention, most commonly the Nuss procedure or modified Ravitch repair [4].

The etiology of PE remains incompletely understood, though substantial evidence points to a genetic component. Familial clustering has been documented in 25–43% of cases, with first-degree relatives of affected individuals showing significantly elevated risk [5,6]. Twin studies and family pedigree analyses suggest complex inheritance patterns that do not follow simple Mendelian genetics, indicating a multifactorial etiology involving multiple genetic loci and potential environmental modifiers [7]. The frequent co-occurrence of PE with heritable connective tissue disorders—including Marfan syndrome (60–70% prevalence), Ehlers–Danlos syndrome, and Poland syndrome—further supports genetic contributions [8,9].

Despite evidence for heritability, the molecular genetic architecture of isolated PE remains largely uncharacterized. Unlike other congenital anomalies with well-defined genetic bases, PE has not been subjected to large-scale genome-wide association studies (GWAS), which have revolutionized understanding of complex trait genetics [10]. The absence of GWAS data creates a significant barrier to several important research avenues: identification of common genetic variants contributing to PE susceptibility; construction of genetic instruments for Mendelian randomization studies to investigate causal relationships between PE and associated conditions; development of genetic risk prediction models; and elucidation of biological pathways amenable to therapeutic targeting [11,12]. Recent advances in next-generation sequencing technologies, including whole-exome sequencing (WES) and whole-genome sequencing (WGS), have enabled family-based genetic studies that have begun to identify candidate genes [13]. Concurrently, mouse models with chest wall phenotypes resembling PE have implicated specific developmental pathways [14]. However, these findings have not been integrated systematically, and their relevance to population-level genetic architecture remains unclear.

PE is increasingly recognized as a complex, multifactorial chest wall deformity in which genetic susceptibility interacts with mechanical, hormonal, nutritional, and epigenetic factors [1,3,7]. While familial clustering and associations with connective tissue disorders support a genetic basis, current evidence indicates that no single gene accounts for disease expression. Variants in genes related to cartilage structure and signaling pathways, such as *COL5A1*, *ACAN*, and *SMAD4* within the TGF-β pathway, appear to function collectively [3,5,8]. These pathways intersect with BMP, FGF, and Wnt signaling to regulate chondrocyte differentiation, cartilage stiffness, and growth synchrony. The typical manifestation or worsening of PE during the pubertal growth spurt underscores the significance of growth velocity and mechanical loading, as genetically susceptible cartilage may deform under repetitive respiratory forces or altered biomechanics. The predominance of PE in males suggests a role for hormonal modulation, with androgens, growth hormone, and the IGF-1 axis potentially amplifying genetic effects on skeletal growth and matrix metabolism [1,14]. In contrast, earlier estrogen-mediated growth plate closure in females may confer protection. Nutritional factors, including vitamin D and micronutrients necessary for collagen synthesis, may reveal latent genetic vulnerabilities during key developmental periods [2,7,12]. Epigenetic mechanisms may also account for the variable expressivity and incomplete penetrance observed within families. Elucidating these gene–gene and gene–environment interactions is essential for refining etiological models, identifying modifiable risk factors, and developing preventive or disease-modifying strategies [12,13,14]. Therefore, integrated genomic and systems biology approaches are critical for advancing PE research and clinical management.

This systematic review identified and characterized all reported genetic associations with pectus excavatum, critically appraised the quality of available genetic evidence, and synthesized findings to highlight candidate genes and biological pathways. The review also identified critical knowledge gaps to inform future genetic studies and advance understanding of pectus excavatum pathogenesis.

## 2. Methods

### 2.1. Search Strategy and Information Sources

We conducted a systematic literature search following Preferred Reporting Items for Systematic Reviews and Meta-Analyses (PRISMA) guidelines [15]. The PRISMA checklist is available as a Supplementary File (S1). This study was also registered with PROSPERO with an ID: CRD420261286190. All electronic databases were searched from inception through 22 November 2025: Google Scholar, PubMed/MEDLINE, Web of Science, SciSpace research database, Embase, and arXiv preprint server. The search strategy was designed in consultation with a medical librarian and piloted to ensure comprehensive retrieval. Further, the same search strategy was utilized consistently across all electronic databases, online repositories (ClinVar, OMIM), and thesis or dissertation repositories. We used Medical Subject Headings (MeSH) for advanced searches, including thesis and dissertation repositories and preprint servers, as well as manual screening of reference lists from key articles. This approach helped identify additional eligible studies not captured by electronic database searches, which is especially important in rare-disease genetics.

### 2.2. Search Terms and Boolean Operators

The search strategy employed both Medical Subject Headings (MeSH) terms and free-text keywords combined with Boolean operators. The core search string was structured as follows: Primary search: (“pectus excavatum” OR “funnel chest” OR “chest wall deformity”) AND (“genetic” OR “gene” OR “SNP” OR “single nucleotide polymorphism” OR “polymorphism” OR “GWAS” OR “genome-wide association” OR “exome sequencing” OR “whole genome sequencing” OR “familial” OR “hereditary” OR “variant” OR “mutation”). Secondary searches were conducted for specific genetic concepts: - (“pectus excavatum”) AND (“beta coefficient” OR “odds ratio” OR “effect size” OR “allele frequency”) - (“pectus excavatum”) AND (“Mendelian randomization” OR “genetic instrument” OR “instrumental variable”) - (“pectus excavatum”) AND (“collagen” OR “cartilage” OR “connective tissue” OR “extracellular matrix”). For PubMed, the search was refined using field tags: (pectus excavatum) AND (genetic OR SNP OR polymorphism OR GWAS OR genome-wide). Boolean operators (AND, OR) and truncation symbols (*) were adapted to the specific syntax requirements of each database whilst maintaining semantic equivalence. No language, date, or publication type restrictions were applied during the initial search. This uniform approach ensured comprehensive and reproducible study retrieval across all sources.

### 2.3. Study Selection Criteria

Inclusion criteria: The study included original research articles, case reports, and case series that reported genetic associations with pectus excavatum. Eligible studies also included family-based genetic studies such as linkage analysis, exome sequencing, and segregation analysis; candidate gene association studies; genome-wide association studies, if available; animal model studies using genetic manipulation to produce PE-like phenotypes; and studies of syndromic pectus excavatum where genetic variants are characterized. Only studies published in English or with an English translation were considered. Both human studies and translational animal models were included. Diagnostic criteria for PE varied across the included studies, reflecting differences in clinical practice and research methods. Approaches included clinical assessment by experienced thoracic surgeons, radiological confirmation with computed tomography (CT), and measurement of the Haller index (HI). The HI is defined as the ratio of the transverse chest diameter to the anteroposterior distance between the sternum and vertebral column, with values above 3.25 typically indicating surgical candidacy. Additional criteria included surgical indication based on cardiopulmonary symptoms or severe cosmetic concerns, and ICD-10 coding (Q67.6) in population-based studies. We extracted diagnostic methods from each study and identified this heterogeneity as a potential source of variability in reported genetic associations.

Exclusion criteria: Papers were excluded according to the following criteria: reviews, editorials, or commentaries lacking original data; studies focusing exclusively on surgical techniques or outcomes without genetic data; studies addressing chest wall deformities other than PE, such as pectus carinatum only; abstracts without full-text availability; studies with insufficient genetic characterization, for example, those reporting only “family history” without molecular data; and duplicate publications of the same cohort.

### 2.4. Screening and Data Extraction

Initial screening was performed by reviewing titles and abstracts. Retrieved articles underwent full-text reviews by two independent reviewers (RR and NI). Disagreements were resolved through discussion and consultation with a third reviewer when necessary. Additionally, inter-reviewer agreement for study inclusion was substantial (Cohen’s κ = 0.89; 95% CI: 0.78–0.96), indicating high concordance. Data extraction utilized a standardized form capturing: study characteristics (authors, year, country, study design, sample size); genetic data (genes identified, variant types, chromosomal locations, rsIDs when available); association measures (odds ratios, beta coefficients, *p*-values, confidence intervals); population characteristics (isolated vs. syndromic PE, age, sex distribution); and study quality indicators.

### 2.5. Quality Assessment

Quality assessment was performed using modified Newcastle–Ottawa Scale (NOS) criteria adapted for genetic association studies [16]. For family-based studies, assessment criteria included representativeness of affected families, ascertainment of family history, variant validation, adequacy of phenotypic characterization and follow-up, and reporting of negative findings. For case–control studies, we evaluated case definition, representativeness of cases, selection of controls, comparability of cases and controls, genotyping quality control, and adjustment for population stratification. Based on total scores, studies were classified as high, moderate, or low quality. Meta-analysis was not performed because no quantifiable genetic effect estimates were available. None of the included studies reported effect sizes, standard errors, or allele frequency data required for quantitative synthesis. We therefore conducted a narrative synthesis, organized by study design (family-based sequencing studies, case reports, candidate gene association studies, and animal models) and by biological pathways. Genes were classified according to their primary biological functions using Gene Ontology annotations and supporting the published literature [17].

### 2.6. Assessment of Mendelian Randomization Suitability

We systematically evaluated whether identified genetic associations met criteria for use as instrumental variables in Mendelian randomization studies [18]. Required data elements included SNP rsID; effect allele and alternative allele; effect size (beta coefficient or odds ratio) with standard error or 95% confidence interval; allele frequency; *p*-value; and sample size. The availability of each element was tabulated by all reported genetic associations.

## 3. Results

**Figure 1:**
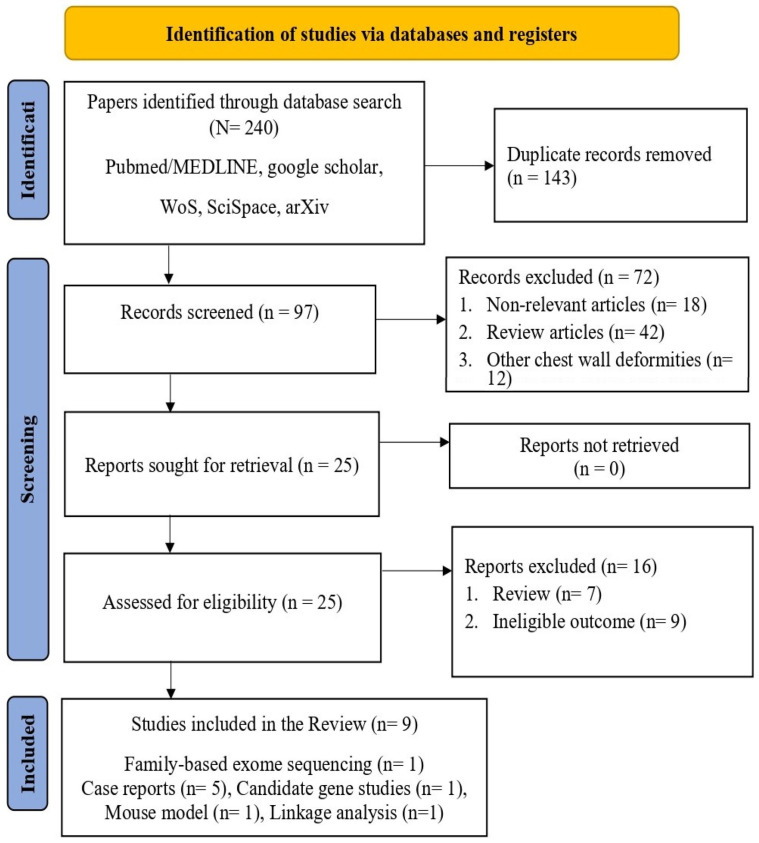
The flow illustrates the different phases of the systematic review according to PRISMA 2020 guidelines [15].

The systematic search identified 240 records across all databases. After removing 143 duplicates, 97 records underwent title and abstract screening. Of these, 72 were excluded (42 reviews and editorials, 18 surgical studies without genetic data, 12 studies of other chest wall deformities). Twenty-five studies underwent full text review, and sixteen were excluded due to ineligible outcomes and study type. No studies were identified that reported genome-wide association analyses for isolated pectus excavatum. Nine studies met the inclusion criteria for this systematic review after full-text screening (Figure 1) [19,20,21,22,23,24,25,26,27]. The included studies comprised family-based exome sequencing studies (n = 1, covering 10 families) [19], case reports (n = 5) [20,21,22,23,24], candidate gene association studies (n = 1) [25], mouse genetic models (n = 1) [26], and linkage analysis studies (n = 1) [27]. Quality assessment revealed substantial heterogeneity. The single family-based exome sequencing study [19] received a high-quality rating (8/9 NOS points) with comprehensive phenotyping, validated variant calling, and appropriate segregation analysis. Case reports scored lower (3–5/9 points) due to small sample sizes and lack of replication. The *ACAN* candidate gene study [25] received moderate quality rating (6/9 points) with adequate case–control design but limited sample size and lack of replication cohort. Mouse model study [26] demonstrated high internal validity with appropriate controls and mechanistic validation.

Family-Based Exome Sequencing: Key Genetic Variants Identified: Fourteen distinct genetic loci were associated with PE (Table 1). No study reported common single nucleotide polymorphisms (SNPs) with rsIDs, effect sizes, or allele frequency data suitable for Mendelian randomization or polygenic risk analysis. Identified variants were rare, family-specific, syndromic, or derived from animal models. A whole-exome sequencing study evaluated 10 unrelated multiplex families, constituting the most comprehensive family-based investigation of isolated PE to date [19]. Segregation analysis identified rare variants in several biologically plausible genes; however, none fulfilled criteria for definitive pathogenicity. *REST* variants identified across multiple families included a heterozygous missense variant (c.70A>G; p.Met24Val) that demonstrated complete segregation with PE. Although in silico predictions indicated tolerance, the recurrence of REST variants across families supports its role as a potential susceptibility gene. *SMAD4*, a mediator of TGF-β/BMP signaling, harbored a promoter variant (c.−69G>A) with complete segregation but low predicted pathogenicity, suggesting a possible modifier effect. *COL5A1* variants, including non-coding regulatory and splice-region changes, exhibited family-specific segregation but were predicted to be benign. All variants were rare (MAF < 0.001), family-specific, and demonstrated incomplete penetrance, consistent with a complex genetic architecture [19]. Notably, effect sizes were not reported.

Case Report Findings: Several case reports identified PE in syndromic or multisystem genetic disorders, predominantly involving connective tissue or RAS/MAPK signaling pathways [20,21,22,23,24]. Two siblings with osteogenesis imperfecta and severe PE carried a copy-number gain involving *COL1A1* and a heterozygous *COL27A1* missense variant (p.Gly697Arg), suggesting digenic mechanisms [20]. A patient with early-onset keratoconus and PE harbored an intronic *COL5A1* splice-region variant [21]. One neurofibromatosis type 1 patient with PE carried a germline *NF1* frameshift variant; a second somatic *NF1* mutation detected exclusively in deformed costal cartilage supported a localized “two-hit” mechanism [22]. Additional reports identified pathogenic variants in *BICD2*, *TGDS*, *SOS1*, *TGFB3* (early-onset PE) [23], and *PTPN11* (Noonan syndrome) [24].

Candidate Gene Association Studies: A population-based case–control genetic association study examined variable number tandem repeat (VNTR) polymorphisms in *ACAN*, which encodes aggrecan, the major proteoglycan of cartilage [25]. Individuals with PE showed a higher frequency of the 27-repeat allele and a lower frequency of the 25-repeat allele compared with controls (*p* < 0.05). However, odds ratios, confidence intervals, and allele frequencies were not reported, and the VNTR-based design precludes standard SNP-based analyses. This study provides limited population-level evidence but remains the only non-familial association analysis in PE.

Animal Model Studies: Karner and colleagues [26] showed loss of cartilage-specific GPR126/Adgrg6 in osteoprogenitor cells in mice develop PE-like dorsal deflection of the sternum along with scoliosis. GPR126 is a G-protein-coupled receptor essential for chondrocyte differentiation. Transcriptomic analysis revealed upregulation of GAL3ST4 (encoding galactose-3-O-sulfotransferase 4) in affected cartilage. While human SNPs near GPR126 have been associated with adolescent idiopathic scoliosis in GWAS, no PE-specific human genetic associations have been reported.

**Figure 2:**
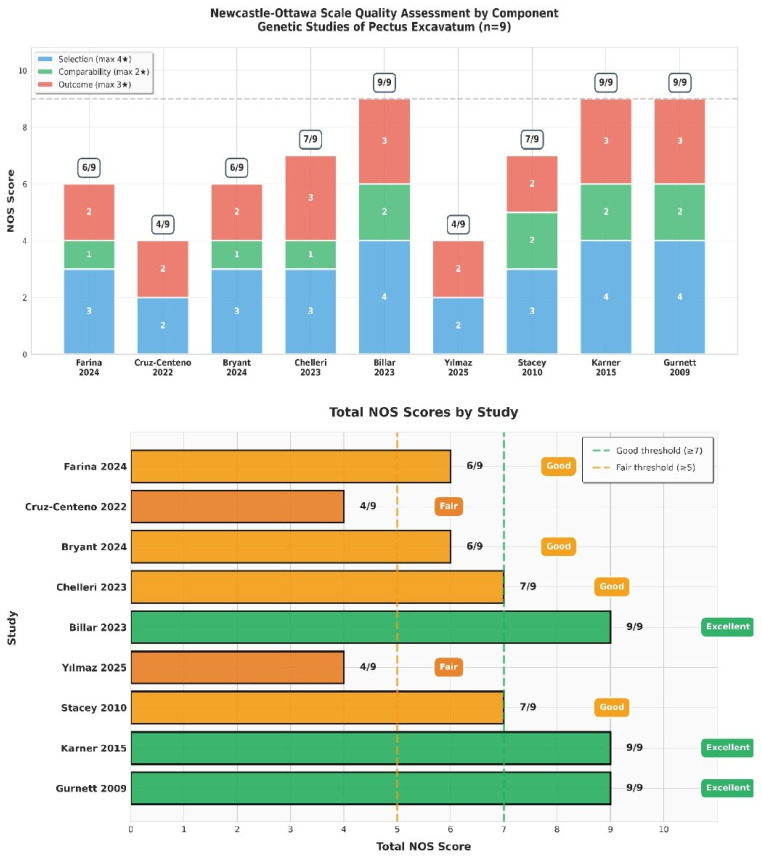
Graphical summary of the Newcastle–Ottawa Scale assessment for included studies [19,20,21,22,23,24,25,26,27], showing low, high, or unclear risk of bias [16].

Assessment of Data Quality and Availability for Mendelian Randomization: Systematic evaluation of all reported genetic associations revealed that none met the criteria for use as instrumental variables in Mendelian randomization studies (Table 2). Critical gaps included absence of common SNP rsIDs (all variants were rare/family-specific), no effect sizes or allele frequencies, lack of genome-wide significant associations (*p* < 5 × 10^−8^), and no independent replication. The single population-level *ACAN* VNTR association [25] was incompatible with standard approaches. However, linkage analysis in a multigenerational family co-segregating PE and scoliosis identified significant linkage at chromosome 18q (logarithm of the odds: LOD 3.86); fine mapping narrowed the interval, but no causative gene emerged [27]. Additionally, Figure 2 summarizes the Newcastle–Ottawa Scale (NOS) assessment for the included studies, indicating the risk of bias as low, high, or unclear. Despite the limited sample size, five studies were classified as good quality [22,23,25,26,27] and four as fair [10,11,12,13,14,15,16,17,18,19,20,21,24] based on the NOS scoring thresholds: scores ≥ 7 are considered good, while scores between 5 and 7 are considered fair.

**Figure 3:**
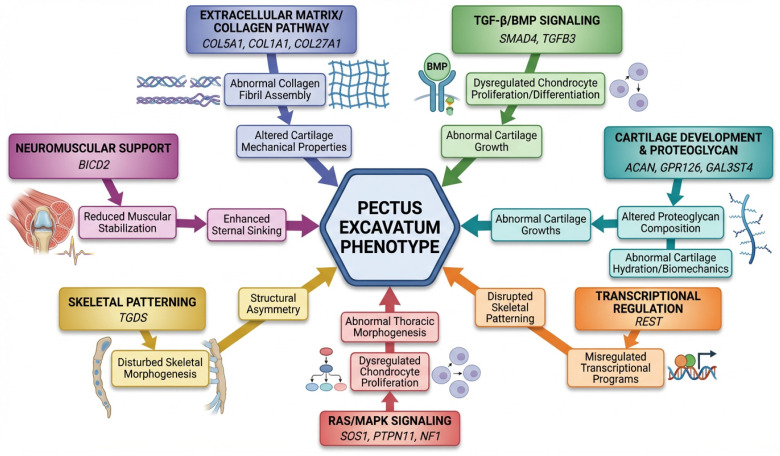
Biological pathways implicated in pectus excavatum pathogenesis [5,6,7,8,30,31,32,33,34,44,45,46].

Biological Pathways Implicated: The identified genes cluster into seven major biological pathways (Figure 3):

1. Extracellular Matrix and Collagen Metabolism (*COL5A1*, *COL1A1*, *COL27A1*): *COL5A1* regulates fibril diameter, which is critical for cartilage ultrastructure [28]. *COL1A1* encodes the primary structural collagen in bone and costal cartilage [29]. *COL27A1* is specific to cartilage and is essential for its structural integrity [30]. Abnormalities in collagen may alter mechanical properties, resulting in abnormal growth and chest wall deformity.
2. TGF-β/BMP Signaling (*SMAD4*, *TGFB3*): *SMAD4* serves as the central mediator in TGF-β and BMP signaling cascades [31], while TGFB3 functions as a key upstream ligand [23]. These pathways regulate chondrocyte proliferation, differentiation, and hypertrophy during endochondral ossification [32]. Dysregulation of these processes has been implicated in skeletal abnormalities [33].
3. Cartilage Development and Homeostasis (*ACAN*, *GPR126*, *GAL3ST4*): Aggrecan confers compressive resistance to cartilage [34]. *GPR126* regulates chondrocyte maturation [26], and *GAL3ST4* modifies proteoglycans [35]. These factors collectively underscore the central role of cartilage biology.
4. Transcriptional Regulation (*REST*): REST acts as a master repressor of neuronal genes in non-neuronal tissues [36] and is implicated in mesenchymal stem cell differentiation [37]. However, its mechanism in pectus excavatum remains unclear.
5. RAS/MAPK Growth-Plate Signaling (*SOS1*, *PTPN11*, *NF1*): This pathway governs chondrocyte proliferation. Hyperactivation in Noonan-spectrum disorders leads to abnormal rib growth and increased chest wall laxity [23,24].
6. Skeletal Patterning (*TGDS*, *COL27A1*, *COL1A1*): Disruptions in thoracic skeletal patterning or altered timing of ossification may increase susceptibility to pectus excavatum [20,23].
7. Neuromuscular Support (*BICD2*): Decreased anterior chest wall support may exacerbate existing structural vulnerabilities [23].

Syndromic Associations: PE is a recognized feature of several Mendelian connective tissue disorders: Marfan syndrome (*FBN1*, 60–70% prevalence) [38,39], Ehlers–Danlos syndrome (multiple genes, particularly *COL5A1* in classical type) [40,41], Noonan syndrome (*PTPN11*, *SOS1*, *RAF1*; 50–70% prevalence) [42,43], and osteogenesis imperfecta (*COL1A1*, *COL1A2*) [44]. Gene overlap between syndromic and isolated PE (particularly *COL5A1*) suggests shared pathogenic mechanisms, with isolated PE potentially representing milder allelic variants or oligogenic combinations.

## 4. Discussion

This systematic review represents the first comprehensive synthesis of genetic associations with pectus excavatum. Our findings reveal a paradoxical situation: while multiple candidate genes have been identified through family studies, case reports, and animal models, the absence of population-level genome-wide association studies leaves fundamental questions about PE genetic architecture unanswered. The identified genes implicate biologically plausible pathways, particularly extracellular matrix biology, cartilage development, and TGF-β signaling—yet the lack of common variant associations and quantitative effect sizes prevents construction of genetic risk models or Mendelian randomization instruments.

### 4.1. Interpretation of Genetic Findings

The convergence of evidence on collagen genes (*COL5A1*, *COL5A2*, *COL1A1*, *COL27A1*) across independent family studies and case reports is notable. Type V collagen, in particular, emerges as a strong candidate, identified in both familial exome sequencing [19] and case reports [21] and known to be mutated in Ehlers–Danlos syndrome classical type, which commonly presents with PE [40]. Type V collagen regulates fibril assembly and is abundant in cartilage [47]. Abnormal type V collagen could plausibly alter the biomechanical properties of costal cartilage, leading to differential growth rates and eventual chest wall deformity. This hypothesis is supported by biomechanical studies showing altered cartilage properties in PE patients [37].

The identification of *SMAD4* variants in familial PE [19] is particularly intriguing given its central role in TGF-β/BMP signaling. These pathways are master regulators of chondrocyte biology [30,31]. Notably, mutations in other TGF-β pathway components cause Loeys–Dietz syndrome, which can include chest wall abnormalities [48]. The *SMAD4* finding suggests that PE may result from dysregulated chondrocyte proliferation or differentiation during the critical period of chest wall development. Future studies should investigate whether PE patients show altered TGF-β signaling in costal cartilage.

The *ACAN* VNTR association [25] is significant as the only population-level genetic association reported for PE. Aggrecan is the major proteoglycan in cartilage, and *ACAN* mutations cause spondyloepiphyseal dysplasia and short stature [45]. The VNTR region influences aggrecan expression levels [46]. However, the lack of replication, small sample size, and absence of quantitative effect estimates limit interpretation. Validation in larger cohorts with SNP-based analyses is needed. The mouse model implicating *GPR126* [26] provides mechanistic insights but requires human validation. While *GPR126* variants associate with adolescent idiopathic scoliosis in human GWAS [41], PE-specific associations have not been tested. Given the co-occurrence of scoliosis and PE [27], *GPR126* represents a priority candidate for future human genetic studies.

### 4.2. Implications for Mendelian Randomization Studies

A key motivation for this review was to assess the feasibility of Mendelian randomization studies investigating causal relationships between PE and associated conditions (e.g., cardiovascular outcomes, psychological effects). This study’s findings definitively demonstrate that MR studies are currently not possible due to the complete absence of genetic instruments. MR requires genetic variants robustly associated with the exposure (PE); variants independent of confounders; and variants affecting the outcome only through the exposure [18]. Without even the first criterion met, MR cannot proceed, which represents a significant research gap. MR has proven powerful for investigating causal relationships in other conditions [49] and could address important questions about PE, such as whether the condition causally affects cardiovascular function or whether observed associations reflect confounding. The inability to conduct MR studies highlights the urgent need for PE GWAS.

### 4.3. The Critical Need for Genome-Wide Association Studies

A key finding of this review is the complete absence of genome-wide association studies (GWAS) focused on isolated pectus excavatum (PE). This absence is particularly notable because PE is relatively common, occurring in approximately 1 in 400 live births [2], exhibits substantial heritability with familial clustering estimates ranging from 25% to 43% [5,6], and can be objectively quantified using established measures such as the Haller index and computed tomography (CT) imaging [50]. Furthermore, GWAS have been successfully conducted for many conditions that are both rarer and less clearly defined than PE [51]. Multiple factors likely contribute to this gap. First, phenotyping is challenging because PE encompasses a clinical spectrum ranging from mild cosmetic deformity to severe cardiopulmonary compromise. However, quantitative indices such as the Haller index provide objective criteria for case definition [50]. Second, large population biobanks may under-ascertain PE due to reliance on International Classification of Diseases (ICD) coding or self-report, although the increasing availability of imaging data may facilitate algorithm-based phenotyping [52]. Third, there is a lack of large, dedicated research cohorts that integrate genetic and detailed phenotypic data for PE. Finally, existing genetic studies are biased toward surgical populations, which may over-represent severe cases and fail to capture milder phenotypes that could have distinct genetic architectures.

To address these barriers, future research should utilize biobank imaging in combination with machine learning-based detection of PE, establish international consortia dedicated to PE, standardize phenotyping protocols, and recruit both surgical and non-surgical cases. Power calculations suggest that approximately 5000 cases and 25,000 controls are necessary to detect common genetic variants with modest effect sizes at genome-wide significance [53], underscoring the importance of collaborative meta-analyses.

### 4.4. Biological Insights and Therapeutic Implications

Whilst we employed a comprehensive multi-database search strategy, the possibility of study omission cannot be entirely excluded, particularly for non-English publications. The heterogeneity in study designs, diagnostic criteria, and phenotypic characterization limits direct comparability of findings. The absence of standardized reporting of genetic variants precluded quantitative synthesis. Our review was limited to published literature, and unpublished data may exist. The lack of independent replication for most reported genetic associations limits confidence in their validity. Despite limitations in population-level data, the convergence on specific biological pathways provides valuable insights. The prominence of collagen and ECM genes suggests that PE fundamentally reflects abnormal connective tissue biology in costal cartilage. This has potential therapeutic implications. If specific collagen abnormalities could be identified in individual patients, targeted interventions might be possible. For example, vitamin C supplementation improves collagen synthesis [54], and TGF-β pathway modulators are in development for other connective tissue disorders [55].

The cartilage development pathway genes (*ACAN*, *GPR126*) suggest that PE results from abnormal cartilage growth during development. Understanding the critical developmental windows and molecular mechanisms could inform timing of interventions. Currently, surgical correction is typically performed in adolescence [4], but if genetic risk could be identified early, earlier intervention or preventive approaches might be considered. The identification of somatic *NF1* inactivation specifically in PE cartilage [22] raises the intriguing possibility that PE in some cases results from somatic mosaicism rather than germline variants. This “two-hit” mechanism is well-established in cancer [56] but less explored in developmental anomalies. If somatic variants contribute substantially to PE, this would have important implications for genetic counseling and recurrence risk estimation.

Comparing PE genetic findings with related chest wall and skeletal conditions provides context. Adolescent idiopathic scoliosis (AIS), which frequently co-occurs with PE [27], has been subjected to multiple GWAS identifying common variants near *GPR126*, *LBX1*, and other loci [41,57]. The shared *GPR126* association between AIS mouse models [26] and human AIS GWAS [41] suggest overlapping genetic architecture. However, PE-specific GWAS are needed to determine whether the same variants contribute to PE or whether distinct genetic factors are involved. Poland syndrome, another chest wall anomaly, also lacks GWAS but has been linked to disruptions in subclavian artery development during embryogenesis [58]. The distinct pathophysiology suggests different genetic underpinnings, though both conditions may involve disrupted developmental pathways. Figure 3 illustrates a proposed biological mechanism involved in the pathogenesis of pectus excavatum. The pectus excavatum phenotype arises from multiple dysregulated pathways. Mutations in extracellular matrix genes (*COL5A1*, *COL1A1*, *COL27A1*) result in abnormal collagen fibril assembly, compromising cartilage mechanical properties [5,30,31,32,33,34,46]. Dysregulation of TGF-β/BMP signaling (*SMAD4*, *TGFB3*) impairs chondrocyte proliferation and differentiation. Variants in cartilage development genes (*ACAN*, *GPR126*, *GAL3ST4*) modify proteoglycan composition and hydration [6,7,8,34,46]. Aberrations in the RAS/MAPK pathway (*SOS1*, *PTPN11*, *NF1*) disrupt chondrocyte proliferation and thoracic morphogenesis. Furthermore, BICD2 influences neuromuscular support, *TGD5* alters skeletal patterning, and *REST* misregulates transcriptional programs [5,8,30,44,46]. Collectively, these factors contribute to structural asymmetry and sternal depression.

### 4.5. Syndromic Versus Isolated PE

The co-occurrence of PE with recognized syndromic conditions offers significant insights into its underlying biology and raises fundamental questions regarding the genetic basis of isolated PE [59,60,61,62]. One explanation is the allelic spectrum model, in which hypomorphic variants in genes typically associated with syndromic disorders result in isolated chest wall deformity while more severe loss-of-function variants produce full syndromic presentations. This model is exemplified by genes such as *FBN1*, where mild variants may cause isolated PE without meeting diagnostic criteria for Marfan syndrome. Incomplete penetrance and variable expressivity may also account for cases in which carriers of pathogenic variants present only with PE, a phenomenon frequently observed in connective tissue disorders and likely influenced by genetic modifiers, epigenetic regulation, and environmental factors [60,61,62,63]. Alternatively, the polygenic overlap model suggests that isolated PE arises from the cumulative effect of multiple common variants of small effect within pathways disrupted by rare variants in syndromic cases, such as those involved in collagen metabolism, TGF-β signaling, and cartilage development. Isolated PE may also result from distinct genetic mechanisms, with variants affecting localized chest wall development without systemic involvement, as supported by loci on chromosome 18q and genes such as *REST*, which are not typically associated with known syndromes [60,64]. Comparative analyses demonstrate shared features between syndromic and isolated PE, including male predominance, familial clustering, and variable severity, as well as notable differences such as later presentation and absence of extra-thoracic manifestations in isolated cases. These distinctions have important clinical and research implications, highlighting the need for thorough phenotypic assessment, appropriate genetic counseling, and genome-wide study designs to clarify shared and distinct genetic architectures [59,60,61,62]. A major limitation in genetic studies of PE is the substantial heterogeneity in phenotype definition and severity assessment. PE spans a spectrum from mild cosmetic deformity to severe cardiopulmonary compromise, yet diagnostic thresholds differ across studies, ranging from subjective visual assessment to quantitative measures such as Haller index > 3.25. This variability may capture different genetic architectures, while surgical cohorts risk over-representing syndromes [63,64]. Inconsistent documentation of symmetry-associated skeletal or connective tissue features, and severity grading, further limit genotype–phenotype correlation. Standardized diagnostic criteria, severity scales, and comprehensive phenotyping are therefore essential to improve reproducibility and interpretability of genetic findings [60,61,62,63,64,65].

### 4.6. Limitations of Current Evidence

This review is subject to several limitations. Restricting inclusion criteria to English-language publications may have led to the omission of relevant studies. Grey literature was not systematically searched, and unpublished data were not requested from study authors. While quality assessment tools were adapted for genetic studies, these instruments may not fully capture methodological nuances across diverse study designs. Quantitative meta-analysis was not feasible due to the absence of comparable effect estimates and substantial heterogeneity among included studies, such as family-based sequencing, case reports, candidate gene studies, and animal models. Human genetic studies in this field have consistently been limited in scale; for example, the largest family-based study included only 10 families [19], and most reports involved one to three individuals. The *ACAN* study [25] provided limited population-level data with incomplete reporting, which substantially restricted statistical power and generalizability. Furthermore, most included studies consisted of case reports or small family-based investigations lacking independent replication, which reduces confidence in the validity and generalizability of the reported genetic associations. The lack of functional validation for most variants further limits mechanistic interpretation and weakens causal inference. All identified variants were rare (MAF < 0.001) and often family specific. Although rare variants may exert large effects, the contribution of common variants, typically identified through genome-wide association studies (GWAS), remains unexplored. Publication bias likely inflates the perceived strength of evidence, as null findings are rarely published. Most variants lack experimental functional validation and rely on in silico predictions, which are insufficient to establish causality [19]. Although we have strengthened the findings by explicitly detailing the phenotypic features observed in the GPR126 and GAL3ST4 mouse models and their potential relevance to PE, the translational applicability of these models to human isolated PE remains limited, as supporting evidence is scarce. Furthermore, all studies were conducted in European or East Asian populations, thereby limiting the generalizability of the findings to other populations.

### 4.7. Recommendations for Future Research

Immediate priorities for the next one to two years include establishing an international pectus excavatum (PE) consortium with standardized phenotyping, utilizing existing biobanks (such as UK Biobank, FinnGen, and All of Us) through ICD codes and imaging data, and developing machine learning algorithms for automated PE detection from chest CT or MRI scans. Essential tasks also include targeted sequencing of candidate genes (*COL5A1*, *COL5A2*, *SMAD4*, *ACAN*) in large case–control cohorts and replication of the *ACAN* VNTR association [25]. Medium-term priorities (two to five years) encompass adequately powered GWAS (at least 5000 cases and 25,000 controls) with meta-analysis across cohorts, functional validation using patient-derived chondrocytes or induced pluripotent stem cell (iPSC) models, and investigation of gene–environment interactions, somatic mosaicism, and tissue transcriptomics or proteomics. Long-term priorities (five to ten years) include developing polygenic risk scores [12], investigating oligogenic mechanisms, conducting causal inference studies, and translating genetic insights into clinical risk stratification, syndromic screening, recurrence counseling, and preventive or therapeutic strategies for pectus excavatum [65,66]. Beyond traditional genome-wide association studies (GWAS), several emerging genomic approaches may enhance understanding of pectus excavatum (PE), yet significant limitations remain. Polygenic risk scores require large, ancestrally diverse cohorts and currently lack clinical validation for PE [12,23,65]. Analyses of structural and rare variants rely on costly whole-genome sequencing and robust analytical pipelines, which limit scalability. Interpretation of non-coding regulatory variants is constrained by tissue and developmental stage specificity, as well as the limited availability of relevant cartilage datasets [66,67,68]. Multi-omics and single-cell approaches are resource-intensive and remain primarily exploratory. Consequently, the integration of these methods into clinical decision-making and precision interventions is not yet feasible.

## 5. Conclusions

This systematic review reveals a critical gap between established PE heritability and available population-level genetic data. Whilst family-based studies implicate *COL5A1*, *COL5A2*, *SMAD4*, and *ACAN* in collagen metabolism, cartilage development, and TGF-β signaling, no GWAS exists, precluding comprehensive understanding of genetic architecture and causal inference via Mendelian randomization. International collaborative GWAS leveraging biobank resources with standardized phenotyping are urgently needed to elucidate PE’s molecular basis, enabling precision risk prediction, mechanistic insights, and therapeutic development.

## Figures and Tables

**Table 1:** Genetic loci associated with pectus excavatum [19,20,21,22,23,24,25,26,27].

Gene	Chromosome	Study Type	Sample Size	Variant Type	Biological Function
*REST* [19]	4q12	Family exome sequencing	10 families, 10 cases	Missense variant (c.70A>G; p.Met24Val)	Transcriptional repression
*SMAD4* [19]	18q21.2	Family exome sequencing	10 families, 10 cases	Promoter-region variant (c.-69G>A)	TGF-β/BMP signaling
*COL5A1* [19,21]	9q34.2-q34.3	Family exome sequencing; Case report	10 families; 10 patients	5′UTR regulatory variant; intronic splice-altering variant	Type V collagen, extracellular matrix
*COL1A1* [20]	17q21.33	Case report	2 siblings	Copy number gain	Type I collagen, extracellular matrix
*COL27A1* [20]	9q32	Case report	2 siblings	Missense variant (p.Gly697Arg)	Type XXVII collagen, cartilage ECM organization and cartilage-to-bone transition
*NF1* [22]	17q11.2	Case report	1 case	Germline and somatic frameshift variants	Tumor suppressor; RAS/MAPK pathway
*BICD2* [23]	9q22.31	Cohort study	11 cases	Pathogenic variant (neuromuscular disorder)	Intracellular transport; motor neuron function
*TGDS* [23]	13q32.1	Cohort study	11 cases	Pathogenic variant (Catel–Manzke syndrome)	Skeletal development
*SOS1* [23]	2p22.1	Cohort study	11 cases	Pathogenic variant (Noonan syndrome)	RAS/MAPK signaling
*TGFB3* [23]	14q24.3	Cohort study	11 cases	Pathogenic variant (Loeys–Dietz syndrome)	TGF-β signaling; connective-tissue regulation
*PTPN11* [23,24]	12q24.13	Case report	1 patient	Missense and Pathogenic variant	Protein tyrosine phosphatase; RAS/MAPK signaling
*ACAN* [25]	15q26.1	Case–control	158 cases	VNTR polymorphism (27-repeat allele)	Cartilage proteoglycan
*GPR126* [26]	6q24.1	Mouse model	Mouse	Deletion	Chondrocyte differentiation
*GAL3ST4* [26]	1q42.3	Mouse model	Mouse	Upregulation	Proteoglycan sulfation
Unknown locus [27]	18q	Linkage analysis	1 large family (23 family members)	Linkage peak (LOD score 3.86); no mutation identified	Unknown

VNTR, variable number tandem repeat; LOD, logarithm of the odds.

**Table 2:** Assessment of data availability for Mendelian randomization [19,20,21,22,23,24,25,26,27].

Required Data Element	Available for PE?	Notes
SNP rsID	No	All variants are rare or private to families; no common SNPs reported
Effect allele/Other allele	No	Allelic effects not specified in any study
Beta coefficient or Odds ratio	No	No quantitative effect sizes reported from population studies
Standard error or 95% CI	No	Precision estimates not provided
*p*-value (genome-wide significant)	No	No GWAS conducted; no *p* < 5 × 10^−8^ associations
Effect allele frequency	No	Population-level allele frequencies not reported
Sample size (cases/controls)	No	No population-based case–control studies with genetic data
Independent replication	No	No findings replicated in independent cohorts
MR Feasibility	Not possible	No genetic instruments available

PE, pectus excavatum; SNP, single nucleotide polymorphism; CI, confidence interval; GWAS, genome-wide association study; MR, Mendelian randomization.

## Data Availability

The original contributions presented in this study are included in the article/Supplementary Material. Further inquiries can be directed to the corresponding author.

## Supplementary Materials

The following supporting information can be downloaded at https://www.mdpi.com/article/10.3390/cimb48010122/s1.
